# Metabolic acid-base adaptation triggered by acute persistent hypercapnia in mechanically ventilated patients with acute respiratory distress syndrome

**DOI:** 10.5935/0103-507X.20160009

**Published:** 2016

**Authors:** Thiago Gomes Romano, Mario Diego Teles Correia, Pedro Vitale Mendes, Fernando Godinho Zampieri, Alexandre Toledo Maciel, Marcelo Park

**Affiliations:** 1 Department of Nephrology, Faculdade de Medicina do ABC - Santo André (SP), Brazil.; 2 Intensive Care Unit, Hospital Sírio Libanês, São Paulo, Brazil.; 3 Intensive Care Unit, Emergency Department, Hospital das Clinicas, Faculdade de Medicina, Universidade de São Paulo - São Paulo (SP), Brazil.

**Keywords:** Acidosis, respiratory, Respiratory distress syndrome, adult, Acid-base equilibrium, Respiration, artificial, Intensive care units

## Abstract

**Objective:**

Hypercapnia resulting from protective ventilation in acute respiratory distress syndrome triggers metabolic pH compensation, which is not entirely characterized. We aimed to describe this metabolic compensation.

**Methods:**

The data were retrieved from a prospective collected database. Variables from patients' admission and from hypercapnia installation until the third day after installation were gathered. Forty-one patients with acute respiratory distress syndrome were analyzed, including twenty-six with persistent hypercapnia (PaCO_2_ > 50mmHg > 24 hours) and 15 non-hypercapnic (control group). An acid-base quantitative physicochemical approach was used for the analysis.

**Results:**

The mean ages in the hypercapnic and control groups were 48 ± 18 years and 44 ± 14 years, respectively. After the induction of hypercapnia, pH markedly decreased and gradually improved in the ensuing 72 hours, consistent with increases in the standard base excess. The metabolic acid-base adaptation occurred because of decreases in the serum lactate and strong ion gap and increases in the inorganic apparent strong ion difference. Furthermore, the elevation in the inorganic apparent strong ion difference occurred due to slight increases in serum sodium, magnesium, potassium and calcium. Serum chloride did not decrease for up to 72 hours after the initiation of hypercapnia.

**Conclusion:**

In this explanatory study, the results indicate that metabolic acid-base adaptation, which is triggered by acute persistent hypercapnia in patients with acute respiratory distress syndrome, is complex. Furthermore, further rapid increases in the standard base excess of hypercapnic patients involve decreases in serum lactate and unmeasured anions and increases in the inorganic apparent strong ion difference by means of slight increases in serum sodium, magnesium, calcium, and potassium. Serum chloride is not reduced.

## Abstract (pt)

**Objetivo:**

A hipercapnia resultante da ventilação protetora na síndrome do desconforto respiratório agudo desencadeia uma compensação metabólica do pH que ainda não foi completamente caracterizada. Nosso objetivo foi descrever esta compensação metabólica.

**Métodos:**

Os dados foram recuperados a partir de uma base de dados registrada de forma prospectiva. Foram obtidas as variáveis dos pacientes no momento da admissão e quando da instalação da hipercapnia até o terceiro dia após sua instalação. Analisamos 41 pacientes com síndrome do desconforto respiratório agudo, incluindo 26 com hipercapnia persistente (pressão parcial de gás carbônico acima de 50mmHg por mais de 24 horas) e 15 sem hipercapnia (Grupo Controle). Para a realização da análise, utilizamos uma abordagem físico-química quantitativa do metabolismo acidobásico.

**Resultados:**

As médias de idade dos Grupos com Hipercapnia e Controle foram, respectivamente, de 48 ± 18 anos e 44 ± 14 anos. Após a indução da hipercapnia, o pH diminuiu acentuadamente e melhorou gradualmente nas 72 horas seguintes, de forma coerente com os aumentos observados no excesso de base padrão. A adaptação metabólica acidobásica ocorreu em razão de diminuições do lactato sérico e do *strong ion gap* e de aumentos na diferença aparente de *strong ions* inorgânicos. Além do mais, a elevação da diferença aparente de *strong ions* inorgânicos ocorreu por conta de ligeiros aumentos séricos de sódio, magnésio, potássio e cálcio. O cloreto sérico não diminuiu por até 72 horas após o início da hipercapnia.

**Conclusão:**

A adaptação metabólica acidobásica, que é desencadeada pela hipercapnia aguda persistente em pacientes com síndrome do desconforto respiratório agudo, foi complexa. Mais ainda, aumentos mais rápidos no excesso de base padrão em pacientes com hipercapnia envolveram diminuições séricas de lactato e íons não medidos, e aumentos na diferença aparente de *strong ions* inorgânicos, por meio de ligeiros aumentos séricos de sódio, magnésio, cálcio e potássio. Não ocorreu redução do cloreto sérico.

## INTRODUCTION

Acute respiratory distress syndrome (ARDS) is a common reason for initiating ventilatory support in critically ill patients.^(1)^ ARDS mortality remains as high as 54% despite advances in critical care.^(2)^ To minimize ventilator-induced lung injury,^(3)^ a protective mechanical ventilation based on lower tidal volumes and lower distention pressures is recommended.^(4)^

Lung injury increases dead space ventilation.^(5)^ Furthermore, protective ventilation is associated with reduced effective alveolar ventilation; both factors result in ineffective carbon dioxide clearance from the blood, resulting in hypercapnia.^(6)^ Currently, there is no consistently demonstrated clinical benefit of hypercapnia in ARDS patients.^(7)^

Protective ventilation-induced hypercapnia evokes metabolic responses toward pH normalization within a short period.^(6)^ In patients with chronic hypercapnic hypoventilation, the metabolic acid-base adaptation is related to plasmatic bicarbonate (HCO_3_) elevation and chloride reduction.^(8)^ Normal subjects who are acutely exposed to hypercapnia exhibit increased urinary chloride elimination.^(9)^ In contrast, critically ill patients commonly present with reduced renal chloride removal.^(10,11)^ Therefore, the mechanism of pH compensation in hypercapnic patients with ARDS has not been established. The aim of this study was to explore the mechanisms involved in pH compensation during acute hypercapnia in patients with ARDS for at least 24 hours.

## METHODS

The Ethical Committee of the *Hospital das Clínicas* of *Faculdade de Medicina* of the *Universidade de São Paulo* approved this study (approval document number 107.443), and informed written consent was waived. Patient records/information was anonymized and de-identified prior to analysis.

We retrospectively reviewed electronic medical records that were prospectively collected from 1275 patients who were consecutively admitted to our intensive care unit (ICU) from June 2007 to June 2012.

The inclusion criteria were bilateral pulmonary infiltrates on the X-ray, acute onset of hypoxemia, P/F ratio < 300mmHg using a *positive end-expiratory pressure* (PEEP) ≥ 5cmH_2_O, and no cardiogenic cause of the pulmonary infiltrate.

The exclusion criteria were chronic renal failure on dialysis support, acute kidney injury with any renal replacement therapy mode necessity, bicarbonate infusion and chronic hypercapnia.

The patients were categorized according to the presence or lack of persistent acute hypercapnia. Persistent acute hypercapnia was defined as a partial pressure of carbon dioxide (PaCO_2_) greater than 50mmHg for more than 24 hours, with at least three arterial blood gas sample analyses during this period, in patients with documented previous normal PaCO_2_ values and without a history of chronic hypercapnia. The hypercapnic group was compared with the control group in order to explore the metabolic compensation to the hypercapnia.

ARDS was defined according to the Berlin conference.^(12)^ The information that was obtained from the patients' charts included the following: demographic characteristics (age, gender, weight, height and co-morbidities) and ICU data from the time of admission until the third day after hypercapnia diagnosis [respiratory failure etiology, expected mortality (calculated by the Acute Physiology and Chronic Health disease Classification System - APACHE II)]^(13)^ or the simplified acute physiological score (SAPS 3),^(14)^ sequential organ failure assessment (SOFA)^(15)^ score on the first day of ICU stay, need for vasopressors and/or inotropics, laboratory data, fluid balance, diuresis, and daily variations in heart rate, respiratory rate, temperature and mean arterial pressure.

Acid-base arterial blood samples from the day of ICU admission and one day before the hypercapnia installation, with three samples every 8 hours on the day of hypercapnia installation and up to three days after hypercapnia initiation, were analyzed as well as clinical and physiological daily data from these same days. We emphasize that all of the patients were admitted with the diagnosis of ARDS. The laboratorial data that were retrieved during the hypercapnia installation were routinely collected at least every 8 hours until PaCO_2_ stabilization (generally considered when the PaCO_2_ variation is < 3 - 5%). In the control group, samples were collected at admission and after 24, 48, 72 and 96 hours.

A quantitative physicochemical approach was used to analyze the acid-base variables.^(16)^ In this approach, after several adaptations,^(17)^ the [H^+^] concentration and, hence, pH, were determined using five independent variables: inorganic apparent strong ion difference (SIDai), strong ion gap (SIG), lactate, weak acids in plasma (Atot), and PaCO_2_ variation.^(18)^ The standard equations that were used in this study were the following:

SIDaimEq/L=K+mEq/L+Ca2+mEq/L+Mg2+mEq/L−Cl−mEq/L

SIGmEq/L=SIDai−2.46×10pH−8×PaCO2mmHg+albuming/L×0.123×pH−0.631+phosphatemmol/L×0.309×pH−0.469−lactatemEq/L

Atotg/dL=Albuming/dL+phosphateg/dL

A positive SIG value represents the presence of unmeasured anions, which must be included to determine the measured pH. The standard base excess (SBE) was used in our study to diagnose and quantify the metabolic acid-base variations.^(18)^ The source of SBE variations was analyzed based on SIDai, SIG, lactate, albumin, and phosphate variations.^(18)^

### Statistical analysis

The data distribution was analyzed using the Shapiro-Wilk goodness-of-fit model. The qualitative data, which are shown as occurrences and percentages, were analyzed using Fisher's exact test or chi-square test as appropriate. The quantitative data are presented as the mean and standard deviation values or the medians [25^th^ percentile and 75^th^ percentile], depending on whether the values are parametric or non-parametric, respectively.

The quantitative baseline data were analyzed using non-paired *t*-test or Mann-Whitney's test as appropriate. The quantitative data of two groups over time were analyzed using interaction analyses and a mixed generalized model with the patient as a random factor for determining the within-subject correlation among repeated observations. The Markov chain Monte Carlo procedure using 1000 simulations to obtain the equilibrium of distributions was used to reach a fixed likelihood of each resulting independent variable. The post hoc analyses for interactions were performed using Mann-Whitney's or Wilcoxon's tests as appropriate. The R free open-source statistical package and Comprehensive-R Archive Network (CRAN)-specific libraries were used to build the graphics and to perform all of the statistical analyses.^(19)^

## RESULTS

**Figure 1:**
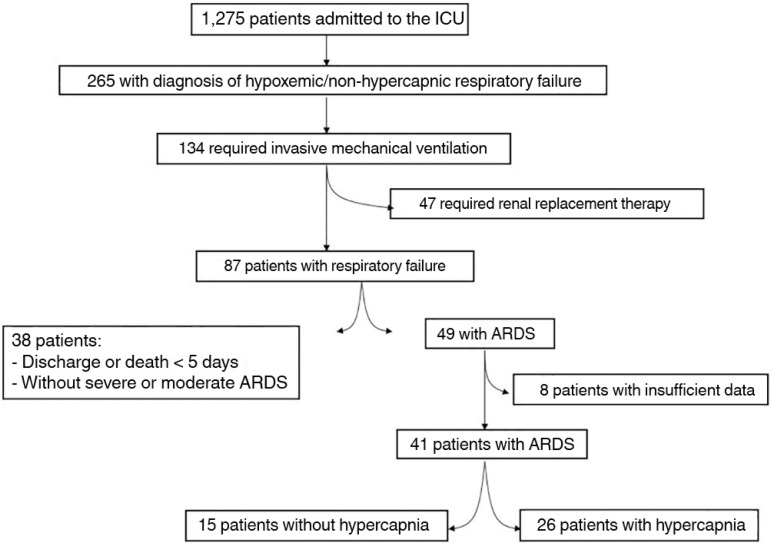
Flowchart of the study. The patients' data were collected from June 2007 to June 2012. ICU - intensive care unit; ARDS - acute respiratory distress syndrome.

There were 49 (4%) patients out of 1275 ICU admissions with ARDS who did not require renal replacement therapy during the study period. A total of 41 patients had the necessary data and were enrolled in the analysis: 26 (64%) patients who developed hypercapnia and 15 (36%) who did not develop hypercapnia as the control group (Figure 1). No patients received diuretics.

**Figure 2:**
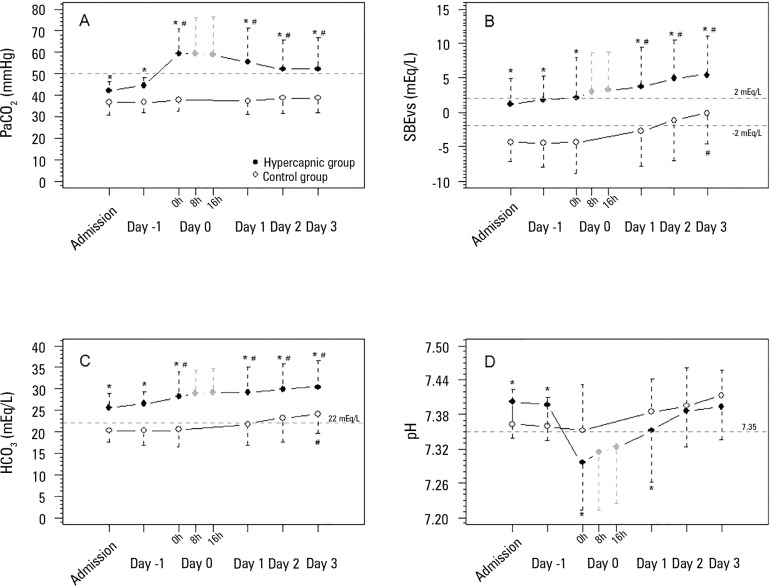
Acid-base variables before and after hypercapnia initiation. A) PaCO_2_ evolution (mixed model fixed effects p = 0.039 for within-group factor analysis, p = 0.009 for between-group factor analysis, and p = 0.119 for group × time interaction analysis). B) Standard base excess evolution (mixed model fixed effects p = 0.077 for within-group factor analysis, p = 0.018 for between-group factor analysis, and p = 0.185 for group × time interaction analysis). C) Bicarbonate evolution (mixed model fixed effects p < 0.001 for within-group factor analysis, p = 0.001 for between-group factor analysis, and p = 0.167 for group × time interaction analysis). D) pH evolution (mixed model fixed effects p = 0.105 for within-group factor analysis, p = 0.008 for between-group factor analysis, and p = 0.219 for group × time interaction analysis). PaCO_2_ - partial pressure of carbon dioxide; SBE - standard base excess; HCO3 - bicarbonate. * Mann-Whitney's post-hoc analysis p < 0.05 versus control group. # Wilcoxon's post-hoc analysis p < 0.05 versus admission day.

The general data for the patients, stratified according to the group, are shown in table 1. The maximum CO_2_ levels in the hypercapnic group occurred within the first 48 hours after the patients' admission, with mean values of 60mmHg. Subsequently, a significant difference between groups was observed up to the third day after hypercapnia commencement (Figure 2).

**Table 1:** General characteristics of the patients in both groups

	**Hypercapnic group**	**Control group**	**p-value**
	**(N = 26)**	**(N = 15)**	
Characteristic			
Age (year)	48 ± 18	44 ± 14	0.364
Gender M/F	15 (58)/11 (42)	5 (33)/10 (67)	0.239
Weight (kg)	56 [50;68]	55 [46;64]	0.600
Height (cm)	164 [158;170]	162 [154;168]	0.655
APACHE II score *	22 [20;24]	18 [16;19]	0.571
SAPS 3 score**	51 [33;53]	33 [26;43]	0.825
SOFA 1^st^ day	5 [3;7]	5 [3;9]	0.999
Comorbidities			0.455
Hypertension	5 (19)	4 (27)	
Diabetes	0 (0)	1 (7)	
COPD	0 (0)	0 (0)	
Chronic renal failure	0 (0)	0 (0)	
Neoplasm	0 (0)	1 (7)	
Respiratory failure etiology			
Pneumonia	22 (84)	13 (87)	1.000
Asthma	2 (8)	0 (0)	0.524
Septic syndromes	2 (8)	2 (13)	0.615
ICU support			
Vasopressors	13 (50)	2 (13)	0.044
Inotropics	11 (42)	6 (40)	0.854
Outcomes			
ICU LOS (days)	11 [6;17]	8 [7;10]	0.118
Mortality	7 (27)	2 (13)	0.445

M/F - male/female; APACHE - acute physiology and chronic health disease classification system; SAPS - simplified acute physiological score; SOFA - sequential organ failure assessment; COPD - chronic obstructive pulmonary disease; ICU - intensive care unit; LOS - length of stay.

* APACHE II score was retrieved from eleven patients;

** SAPS 3 was retrieved from thirty patients. The results expressed in mean and standard deviation values.

Concomitant with the increasing CO_2_, the pH levels decreased, with the lowest values observed on the second day of admission. In the ensuing days, the pH gradually increased to values similar to those observed in the control group. The increases in pH were accompanied by SBE and HCO_3_ elevations, both with a significant difference between the groups (Figure 2). In addition to the SBE elevation, from day 1 to day 3, the PaCO_2_ slightly decreased, remaining greater than 50mmHg.

**Figure 3:**
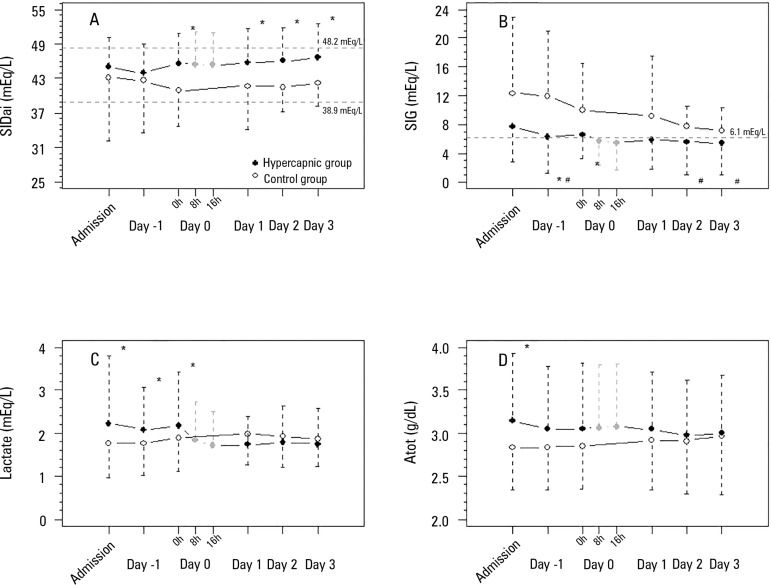
Physicochemical variables of the acid-base metabolic component before and after hypercapnia initiation. A) SIDai evolution (mixed model fixed effects p = 0.646 for within-group factor analysis, p = 0.045 for between-group factor analysis, and p = 0.224 for group × time interaction analysis). B) Strong ion gap evolution (mixed model fixed effects p < 0.001 for within-group factor analysis, p < 0.001 for between-group factor analysis, and p = 0.007 for group × time interaction analysis). C) Lactate evolution (mixed model fixed effects p < 0.978 for within-group factor analysis, p < 0.001 for between-group factor analysis, and p = 0.975 for group × time interaction analysis). D) Atot evolution (mixed model fixed effects p = 0.141 for within-group factor analysis, p = 0.010 for between-group factor analysis, and p = 0.266 for group × time interaction analysis). SIDai - inorganic apparent strong ion difference; SIG - strong ion gap; Atot - weak acids in plasma. * Mann-Whitney's post-hoc analysis p < 0.05 versus control group. # Wilcoxon's post-hoc analysis p < 0.05 versus admission day.

The SIDai was greater in the hypercapnic group and was not accompanied by significant sodium and chloride variations, despite a tendency toward elevated sodium (Figure 3) and figure 1S (http://www.rbti.org.br/content/imagebank/pdf/0103-507X-rbti-28-01-0019-suppl01-en.pdf). The analysis of other physiological and laboratory variables during the observation period demonstrated that the serum hemoglobin, calcium and phosphate levels were different between the two groups (Table 1S in http://www.rbti.org.br/content/imagebank/pdf/0103-507X-rbti-28-01-0019-suppl01-en.pdf). Figure 2S (http://www.rbti.org.br/content/imagebank/pdf/0103-507X-rbti-28-01-0019-suppl01-en.pdf) shows the proportional variation of pH according to the respiratory and metabolic determinants. Figure 1S (http://www.rbti.org.br/content/imagebank/pdf/0103-507X-rbti-28-01-0019-suppl01-en.pdf) shows the main acid-base components of the metabolic adaptation according the patients' disease severity, using an expected mortality of 20% (median of the expected mortalities) as the cut point.

## DISCUSSION

The results of our study show a slight elevation of PaCO_2_ levels in the hypercapnic group before the initiation of hypercapnia, as expected. The SBE and HCO_3_ levels were higher in the hypercapnic group before hypercapnia initiation, and a subsequent increase in HCO_3_ was more relevant than the increase in SBE; however, both the SBE and HCO_3_ levels gradually increased over time (Figure 2) in both groups. The pH initially decreased markedly after hypercapnia initiation and progressively moved toward normalization. The SBE variation in the hypercapnic group could be attributed to an increase in SIDai and a decrease in lactate and in SIG. The SIDai variation was not attributed to chloride variation. In contrast, SIDai improved because of an increase in serum sodium, magnesium and potassium.

Hypercapnia is occasionally required to allow protective ventilation in patients with ARDS. However, the CO_2_ effect *per se* (without the concomitant effect of the tidal overdistention reduction) on lung protection and clinical outcomes is controversial.^(20-23)^ Patients with acute and persistent hypercapnia, in the absence of renal failure, evolve a metabolic compensation toward pH normalization in a short time period (approximately 36 hours).^(6)^ This pH compensation makes the tidal volume reduction more acceptable in patients with ARDS.^(21)^

In our patients, the initial reduced pH promptly moved toward normalization after the initiation of hypercapnia, reaching normal values within a period of 24 - 48 hours. The metabolic response was indicated by HCO_3_ and SBE elevations. The striking initial HCO_3_ elevation most likely occurred because of a stoichiometric factor, i.e., the elevated CO_2_ reacted with water, thereby increasing the HCO_3_ and H^+^.^(24)^ SBE is an independent variable from the acute PCO_2_ variation;^(24)^ thus, a striking initial elevation did not occur in the SBE in our study. The elevation of SBE and its components occurred in both groups during the observed days but was slightly more accentuated in the hypercapnic group during days 1, 2 and 3, when the mean values of SBE were significantly different between groups. The former group presented a less-increased SBE than that of the control group in the admission, probably secondary to the higher PaCO_2_ already at this time in the hypercapnic group.

Exploring the SBE and HCO_3_ elevations after the initiation of hypercapnia, we observed three associated factors in our findings: first, increases in SIDai; second, decreases in lactate; and third, decreases in SIG. The increases in SIDai occurred because of several slight increases in sodium, calcium, magnesium and potassium. We emphasize that serum chloride did not decrease after the initiation of hypercapnia. It is of note that in hypercapnic stable patients with COPD, pH compensation occurred based on a HCO_3_ elevation and chloride reduction in the blood.^(8)^ Renal chloride excretion improved greatly in acutely hypercapnic sheep,^(9)^ indicating the high relevance of chloride modulation in metabolic adaptation triggered by acute hypercapnia in non-critically ill patients. In contrast, in acute critically ill animal models, the acid-base disturbances are marked, especially metabolic acidosis.^(25,26)^ In critically ill patients, metabolic acidosis is relevant, multifactorial, and related to clinical outcomes.^(27,28)^ These patients present reduced sodium and chloride renal excretion,^(10,11,29)^ together with chloride shift from the extravascular to the intravascular compartments added to exogenous load during fluid resuscitation,^(30)^ frequently resulting in hyperchloremia.^(27,31)^ Therefore, we speculate that these factors most likely differentiate the responses of critically ill patients from stable patients when they are exposed to hypercapnia in terms of chloride and SIDai modulations.

The decrease in lactate after the initiation of hypercapnia was another interesting finding in our study. The lactate behavior in hypercapnic patients is consistent with the study of Carvalho et al.^(6)^ In an experimental model of endotoxemia, lactate production decreased with ongoing hypercapnia between 40mmHg and 60mmHg.^(32)^ A similar reduction in lactate production occurred when hypercapnia was initiated in hypoxemic animals.^(33)^ Several mechanisms are related to this lactate-hypercapnia interaction, which involves aerobic mitochondrial metabolism.^(32)^

In our patients, the presence of SIG acidosis at ICU admission can be observed in figure 3 - Panel B. This metabolic acidosis is common in critically ill infected patients, and its improvement during the first five days of ICU stay is associated with better clinical outcomes.^(28)^ This extra source of metabolic acidosis can be a confounder of the pH evolution interpretation.

Unmeasured anion concentrations also decreased after the initiation of hypercapnia. These unmeasured anions have not been identified in humans.^(34)^ In an animal model of hemorrhagic shock, however, these molecules were highly constituted by Krebs cycle components, such as citrate and acetate.^(35)^ Therefore, the aerobic mitochondrial metabolism modulation of hypercapnia may result in SIG variation by the same mechanism of lactate variation. Furthermore, in figure 2S (http://www.rbti.org.br/content/imagebank/pdf/0103-507X-rbti-28-01-0019-suppl01-en.pdf), subtle variations in each metabolic pH determinant can significantly impact pH variation. In this figure, the more parallel the tested variable line is from the x axis, the more striking is its deviation effect on the pH variation, as is, for example, the SIDai.

Clearly, the PaCO_2_ decreased and was associated with the pH normalization during this study in a very important way. This observation probably represents the patient's ventilatory improvement over time. However, metabolic adaptation also occurred, in a similar manner to the control group, but faster.

Our study has many limitations: the data were retrieved from a prospective collected database; different sources of ARDS and sepsis could influence the metabolic adaptation to hypercapnia in different ways; other sources of metabolic acidosis are additional confounders, mainly in two different groups with different disease severities; individual variations were not considered in our study; and this study was drawn only as an explanatory analysis, as there is a paucity of data in this field in the current literature.

## CONCLUSION

In this explanatory study, the results indicate that metabolic acid-base adaptation, which is triggered by acute persistent hypercapnia in patients with acute respiratory distress syndrome, is a complex process. The more rapid standard base excess adaptation than the control group involves decreases in lactate and the strong ion gap and increases in the inorganic apparent strong ion difference, which occur due to slight increases in serum sodium, magnesium, calcium, and potassium but not significant decreases in serum chloride.
