# Supplementary material for: Metabolic acid-base adaptation triggered by acute persistent hypercapnia in mechanically ventilated patients with acute respiratory distress syndrome
Source: Rev Bras Ter Intensiva. 2016 Jan-Mar;28(1):19–26. doi: 10.5935/0103-507X.20160009 (PMC4828087; doi:10.5935/0103-507X.20160009)
Supplement: Supplementary file 1 [file rbti-28-01-0019-suppl01.pdf]

Metabolic acid-base adaptation triggered by acute persistent hypercapnia in mechanically ventilated patients with acute respiratory distress syndrome

Adaptação metabólica diante de hipercapnia persistente aguda em pacientes submetidos à ventilação mecânica por síndrome do desconforto respiratório agudo

Thiago Gomes Romano, Mario Diego Teles Correia, Pedro Vitale Mendes, Fernando Godinho Zampieri, Alexandre Toledo Maciel, Marcelo Park

Table 1S - Other physiological and laboratorial variables of patients during the observational period

Variable	Group	Admission	Day -1	Day 0	Day 1	Day 2	Day 3	p-value
Maximal heart rate (beats/min)	Hypercapnic	115 [111,120]	115 [100,118]	115 [115,120]	115 [114,121]	115 [96,123]	115 [98,128]	0.081 §
	Control	115 [115,128]	115 [110,125]	115 [109,121]	115 [115,115]	115 [115,124]	115 [114,130]	0.149 ¶
Maximal respiratory rate (breaths/min)	Hypercapnic	26 [24,28]	26 [26,28]	26 [26,31]	26 [26,34]	26 [26,37]	27 [26,33]	0.151 §
	Control	26 [24,31]	28 [24,34]	26 [25,36]	26 [25,32]	26 [26,36]	26 [26,36]	0.137 ¶
Maximal ABPm (mmHg)	Hypercapnic	96 [93,96]	96 [90,96]	96 [96,97]	100 [96,117]	101 [96,110]	105 [93,112]	0.380 §
	Control	96 [96,116]	104 [94,112]	103 [88,114]	111 [96,115]	96 [95,121]	110 [96,118]	0.551 ¶
Maximal temperature (ºC)	Hypercapnic	37.0 [36.5,37.9]	37.2 [36.7,37.6]	37 [36.7,37.6]	37 [36.7,37.7]	37 [36.5,37.5]	37 [36.7,37.8]	0.163 §
	Control	37.2 [36.8,37.9]	37.2 [37,37.7]	37.9 [37,37175]	37.2 [36.9,37.7]	37.2 [37,37.5]	37 [36.9,37.5]	0.388 ¶
Cumulative fluid balance (mL)	Hypercapnic	286 [-157,704]	504 [-560,1697]	1225 [-768,3100]	1560 [75,3821]	1889 [35,4191]	1978 [-1635,4421]	0.371 §
	Control	448 [-108,832]	754 [195,1088]	1232 [-307,1816]	1387 [-596,2814]	1574 [-1551,3314]	2134 [-1688,3881]	0.130 ¶
Diuresis (mL)	Hypercapnic	1032 [400,1534]	1210 [941,1770]	1335 # [925,1695]	1025 [776,1484]	1225 [1054,1986]	1155 [918,1776]	0.004 §
	Control	1020 [450,1345]	1240 [695,1569]	1335 [852,2215]	1100 [995,1445]	1900 [1030,2475]	1200 [1030,2515]	0.457 ¶
Total SOFA	Hypercapnic	5 [3,7]	5 [3,7]	4 [2,7]	3 [1,6]	4 [2,7]	3 [1,6]	0.082 §
	Control	5 [3,8]	4 [3,4]	2 [1,4]	4 [2,4]	4 [2,8]	4 [2,7]	0.610 ¶
Hemoglobin (g/dL)	Hypercapnic	10.6 [10.1,11.2]	10.2 [9.3,10.7]	11.0 [8.6,12.8]	9.9 [8.5,12.1]	10.0 * [8.2,12.0]	10.0 [8.0,11.4]	0.135 §
	Control	10.0 [8.5,11.9]	9.5 [8.2,11.1]	9.9 [8.5,11.8]	8.3 [7.7,10.7]	8.7 [7.6,11.1]	8.9 [7.4,11.0]	0.047 ¶
BUN (mg/dL)	Hypercapnic	23 [16,27]	23 # [16,33]	24 # [17,34]	24 # [18,36]	26 # [18,38]	27 # [20,37]	< 0.001 §
	Control	23 [10,34]	24 [11,34]	24 [11,33]	25 [12,46]	22 [16,48]	26 [22,40]	0.625 ¶
Creatinine (mg/dL)	Hypercapnic	0.8 [0.5,0.9]	0.7 [0.5,0.9]	0.7 [0.4,1]	0.7 [0.5,0.8]	0.6 [0.5,0.8]	0.6 [0.4,0.9]	0.140 §
	Control	0.9 [0.7,1.2]	1.0 [0.7,1.6]	0.8 [0.7,1.8]	0.8 [0.7,1.5]	0.8 [0.6,1.3]	0.8 [0.6,1.2]	0.101 ¶
Calcium (mMol/L)	Hypercapnic	1.2 [1.1,1.2]	1.2 # * [1.1,1.2]	1.2 # * [1.1,1.2]	1.2 * [1.1,1.2]	1.2 [1.2,1.2]	1.2 [1.1,1.2]	0.003 §
	Control	1.1 [1.1,1.2]	1.1 [1.1,1.2]	1.1 [1.1,1.2]	1.1 [1.1,1.2]	1.2 # [1.1,1.2]	1.2 # [1.1,1.2]	0.006 ¶
Phosphate (mg/dL)	Hypercapnic	3.7 [2.7,5.3]	3.5 [2.6,4.2]	3.5 * # [2.6,4.2]	2.8 # [2.4,3.7]	3.0 # [2,3.6]	3.2 # [2.4,4.1]	0.034 §
	Control	4.5 [3.2,4.7]	4.3 [3.2,4.7]	4.1 # [2.8,4.7]	3.5 # [2.7,4.7]	3.1 # [2.6,4.5]	3.3 # [2.5,4]	0.017 ¶
Magnesium (mg/dL)	Hypercapnic	1.9 [1.8,2.1]	1.9 [1.8,2.0]	2.1 * # [1.9,2.4]	2.0 # [1.9,2.2]	2.0 [1.9,2.3]	2.1 [1.9,2.3]	< 0.001 §
	Control	1.8 [1.6,2]	1.8 [1.6,2.1]	1.8 [1.6,2.1]	1.8 [1.7,2.2]	2.0 [1.7,2.2]	2 [1.8,2.2]	< 0.001 ¶
Potassium (mMol/L)	Hypercapnic	4.0 [3.4,4.5]	3.7 [3.6,4.1]	4.0 [3.5,4.4]	4.0 * [3.6,4.5]	3.7 [3.3,4.0]	3.7 [3.4,4.0]	< 0.001 §
	Control	3.7 [3.4,4.2]	3.7 [3.4,4.2]	3.6 [3.4,4.1]	3.4 [3.3,3.8]	3.3 # [3.1,3.8]	3.8 # [3.2,4.0]	0.498 ¶
P/F ratio (mmHg)	Hypercapnic	172 [138,218]	248 [196,329]	195 [160,302]	215 [196,277]	229 [160,297]	210 [170,266]	0.084 §
	Control	184 [150,249]	249 [150,303]	234 [146,306]	213 [174,327]	231 [201,376]	249 [176,337]	0.484 ¶

ABPm - arterial blood pressure medium; SOFA - sequential organ failure assessment; BUN - blood urea nitrogen. § p-value mixed model fixed effects for within-group factor analysis; ¶ p-value mixed model fixed effects for between-group factor analysis; * Mann-Whitney's post-hoc analysis $p < 0.05$ versus control group; and # Wilcoxon's post-hoc analysis $p < 0.05$ versus admission.

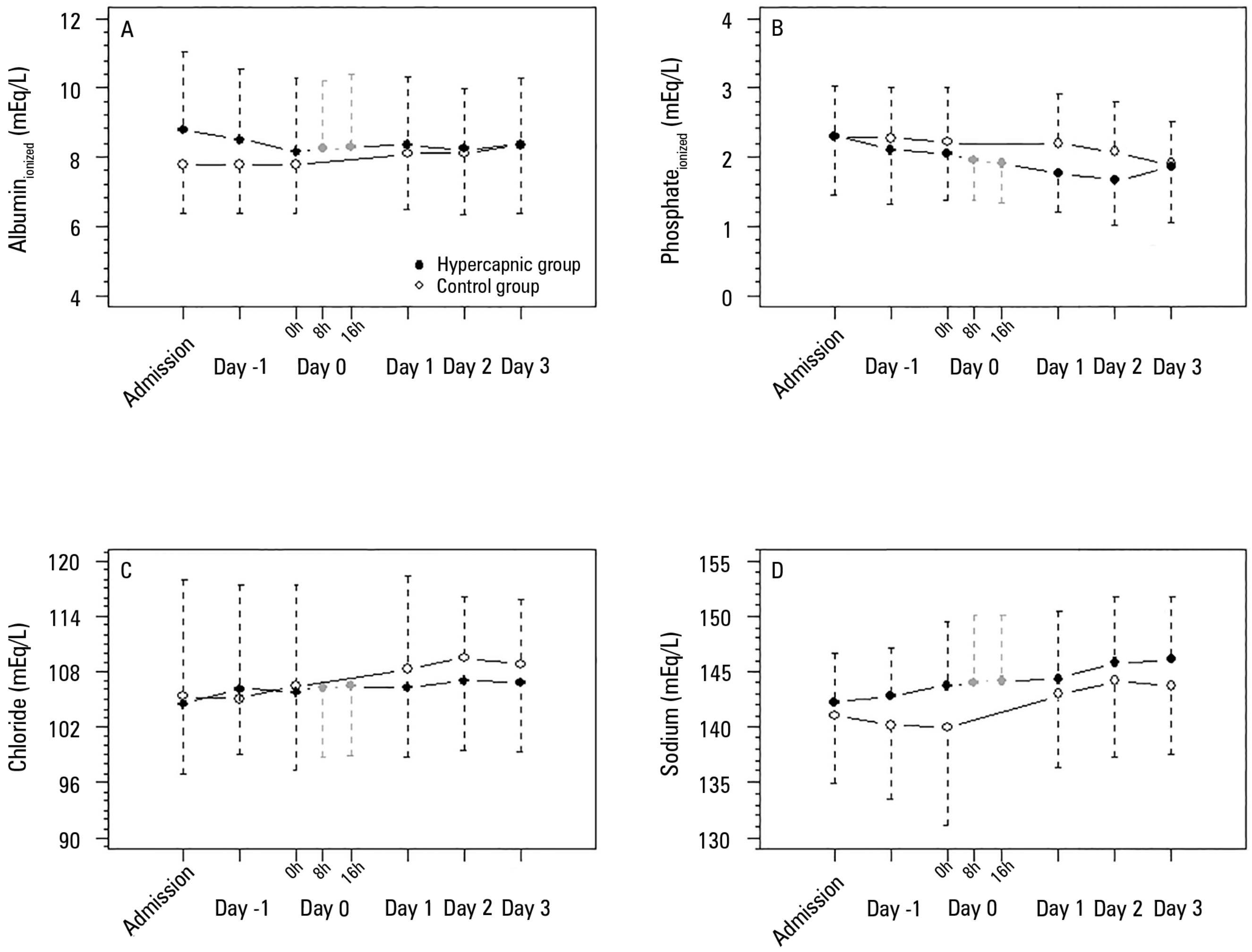

Figure 1S - Physicochemical variables of the acid-base metabolic component before and after hypercapnia initiation. A) Ionized albumin evolution (mixed model fixed effects $p < 0.001$ for within-group factor analysis, $p = 0.021$ for between-group factor analysis, and $p = 0.001$ for group $\times$ time interaction analysis). B) Ionized phosphate evolution (mixed model fixed effects $p = 0.048$ for within-group factor analysis, $p = 0.018$ for between-group factor analysis, and $p = 0.009$ for group $\times$ time interaction analysis). C) Chloride evolution (mixed model fixed effects $p = 0.003$ for within-group factor analysis, $p = 0.744$ for between-group factor analysis, and $p = 0.058$ for group $\times$ time interaction analysis). D) Sodium evolution (mixed model fixed effects $p < 0.001$ for within-group factor analysis, $p = 0.027$ for between-group factor analysis, and $p = 0.186$ for group $\times$ time interaction analysis). * Mann-Whitney's post-hoc analysis $p < 0.05$ versus control group. # Wilcoxon's post-hoc analysis $p < 0.05$ versus admission day.

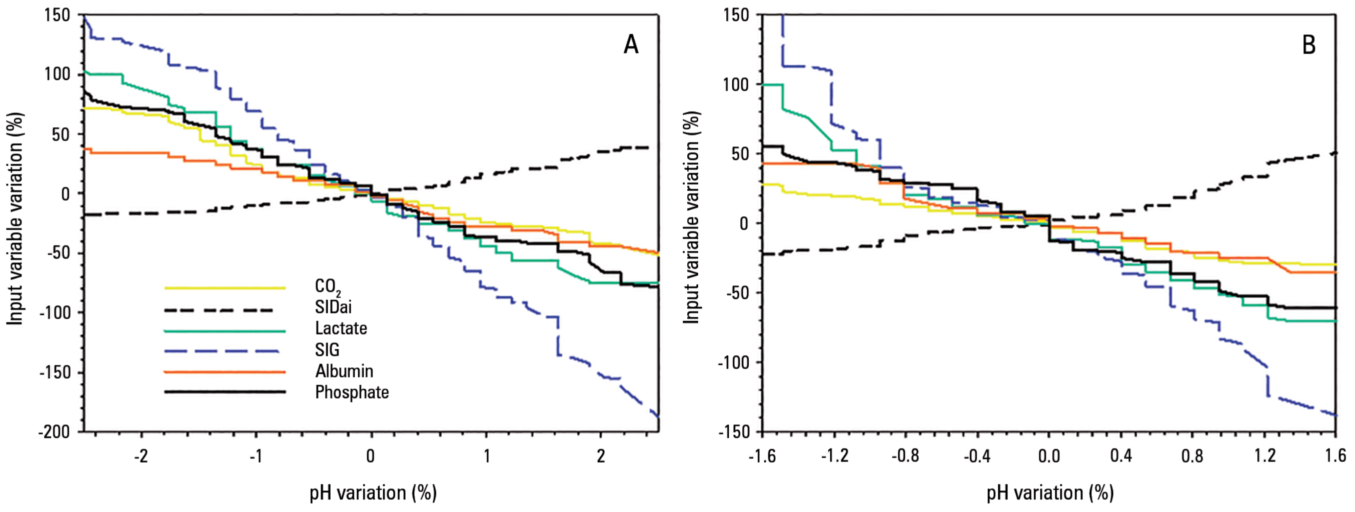

Figure 2S - Spider plots exploring the percentual variation in pH relative to the variations in the respiratory and metabolic determinants of pH. A) Variations in hypercapnic group. B) Variations in the control group. SIDai - inorganic apparent strong ion difference; SIG - strong ion gap.
